# Population dynamics of the sea snake *Emydocephalus annulatus* (Elapidae, Hydrophiinae)

**DOI:** 10.1038/s41598-021-00245-2

**Published:** 2021-10-19

**Authors:** Richard Shine, Gregory P. Brown, Claire Goiran

**Affiliations:** 1 Department of Biological Sciences, Macquarie University, Sydney, NSW 2109 Australia; 2 School of Life and Environmental Sciences, University of Sydney, Sydney, NSW 2006 Australia; 3 LabEx Corail and ISEA, Université de la Nouvelle-Calédonie, BP R4, 98851 Nouméa cedex, New Caledonia

**Keywords:** Ecology, Conservation biology, Evolutionary ecology, Population dynamics, Tropical ecology

## Abstract

For sea snakes as for many types of animals, long-term studies on population biology are rare and hence, we do not understand the degree to which annual variation in population sizes is driven by density-dependent regulation versus by stochastic abiotic factors. We monitored three populations of turtle-headed sea snakes (*Emydocephalus annulatus*) in New Caledonia over an 18-year period. Annual recruitment (% change in numbers) showed negative density-dependence: that is, recruitment increased when population densities were low, and decreased when densities were high. Windy weather during winter increased survival of neonates, perhaps by shielding them from predation; but those same weather conditions reduced body condition and the reproductive output of adult snakes. The role for density-dependence in annual dynamics of these populations is consistent with the slow, K-selected life-history attributes of the species; and the influence of weather conditions on reproductive output suggests that females adjust their allocation to reproduction based on food availability during vitellogenesis.

## Introduction

All populations change in numbers through time, and an understanding of the mechanisms that drive those fluctuations can contribute to management and conservation^1^. Any change in population size from one year to the next may reflect the combined effects of mortality, recruitment, immigration and emigration, each of which may be modified by a range of factors^2^. As a result, the proximate determinants of shifts in abundance are complex and variable in both space and time, even for a single species^3^. One classical paradigm for classifying mechanisms of population regulation invokes a continuum between stochasticity (whereby unpredictable temporal changes in the environment drive major fluctuations in abundance, keeping the population well below carrying capacity) and stability (whereby the population remains close to a consistent upper limit set by finite resource availability)^2,4^. Although no real population conforms exactly to either extreme, the continuum offers a valuable conceptual scheme with which to categorise and compare populations^1,5,6^.

Population fluctuations cannot be measured without long-term data on abundances in the field, encouraging researchers to focus on organisms for which such studies are feasible. Thus, many analyses of population regulation are based on small common insects^5^ or common, large and/or commercially valuable mammals and birds for which detailed records of abundance are available over long periods of time^7,8^. For squamate reptiles, several studies have documented a link between annual variation in population densities and stochastic environmental factors, often mediated by abiotically-driven shifts in prey availability^4,9–12^. For example, annual variation in monsoonal rainfall in the Australian wet-dry tropics affects the availability of several types of prey (mammals, fishes, frogs) and thus the demography of several species of snakes that feed upon those prey^13–16^. However, density-dependence has also been detected in reptile populations, most powerfully through experimental manipulations of abundance^17,18^.

Few data on this topic are available for marine snakes, due to logistical obstacles. Most ecological studies on sea snakes have been based on bycatch from commercial trawlers, or derived from relatively short-term projects^19,20^, and thus cannot address the issue of population regulation. Our long-term (currently, 18-year) annual censuses of turtle-headed sea snakes (*Emydocephalus annulatus*) in New Caledonia provide a unique opportunity to explore this question. Below, we analyse information on numbers and recruitment-relevant traits (age structure, body condition, reproductive rate) to examine the roles of density-dependence and stochastic weather-induced factors in driving year-to-year variation in abundance in sea snake populations in small bays beside the city of Noumea.

## Results

### Changes in abundance through time and space

**Figure 1:**
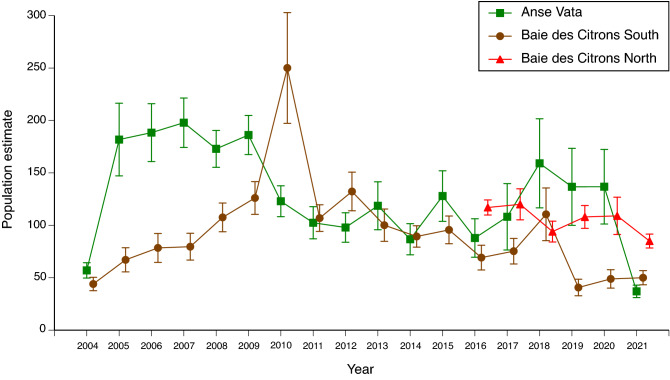
Jolly–Seber estimates of annual population sizes of turtle-headed sea snakes (*Emydocephalus annulatus*) in three study sites near Noumea. Estimates for each year are shown with associated standard errors.

Mean population sizes (densities) were similar among the three study sites (ANOVA with site as the factor, F_2,41_ = 2.88, P = 0.07), but varied through time at each site, with no significant temporal correlation between numerical fluctuations at different sites (the correlation between estimated numbers per year for the two sites with long-term data [Anse Vata vs. Baie des Citrons south] was r^2^ = 0.02, N = 17, P = 0.56; see Fig. 1).

### Density-dependence

Using the method of Dennis and Ponciano^21^, we detected density-dependence in the data from Anse Vata (Likelihood ratio = − 665, P < 0.0001) but not Baie des Citrons south (Likelihood ratio = − 0.65, P = 0.14).

### Correlates of changes in abundance

**Figure 2:**
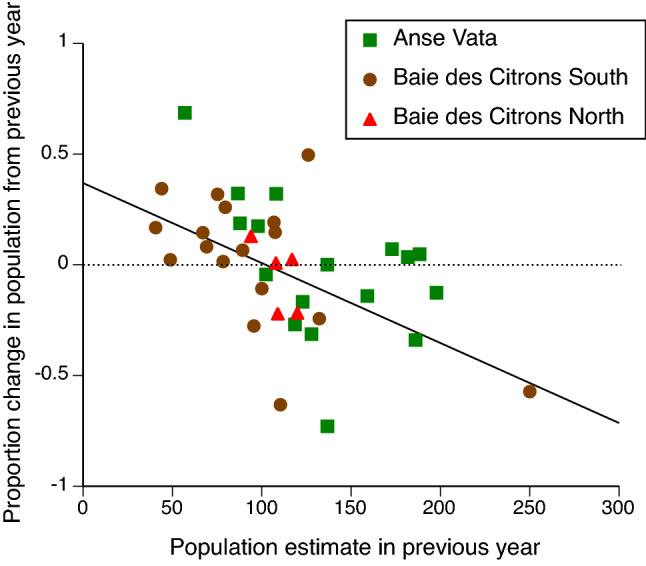
Univariate plot of density-dependence in annual changes in population densities of turtle-headed sea snakes, *Emydocephalus annulatus,* in three populations. Higher population densities were followed by reductions in population density the following year. Dotted horizontal line shows stability (no change in population density from one year to the next), and solid sloping line shows least-squares regression fitted to the combined dataset. See Table 1 for statistical tests of these data, incorporating weather covariates.

Using a mixed-model multiple regression approach, annual changes in abundance showed significant density-dependence; that is, a low population density was generally followed by an increase the following year, whereas a high population density was followed by a decrease (“pop. density” effect in Table 1; see Fig. 2 for univariate plot of these data). Rates of increase in population density were also higher after years when winds were stronger than usual in winter (Table 1).

**Table 1:** Results of statistical analyses of annual changes in population densities (numbers of individuals within 350 × 20 m study areas) of turtle-headed sea snakes (*Emydocephalus annulatus*) in three populations.

Dependent variable	Pop. density	Summer rain	Summer wind	Winter rain	Winter wind
% change in density	F_1,26.72_ = 14.23 **P = 0.001**	F_1,32.09_ = 0.001 P = 0.98	F_1,31.76_ = 2.57 P = 0.12	F_1,32.59_ = 4.00 P = 0.054	F_1,31.86_ = 8.64 **P = 0.006**
Body condition	F_1,30_ = 0.78 P = 0.38	F_1,30_ = 0.55 P = 0.46	F_1,30_ = 0.03 P = 0.87	F_1,30_ = 8.03 **P = 0.008**	F_1,30_ = 9.60 **P = 0.004**
Repro. frequency	F_1,33_ = 0.21 P = 0.64	F_1,33_ = 0.96 P = 0.33	F_1,33_ = 0.64 P = 0.43	F_1,33_ = 0.05 P = 0.82	F_1,33_ = 10.56 **P = 0.003**
Litter size	F_1,33_ = 0.96 P = 0.33	F_1,33_ = 1.12 P = 0.30	F_1,33_ = 0.05 P = 0.83	F_1,33_ = 0.19 P = 0.67	F_1,33_ = 17.90 **P = 0.0002**

Site (population) was included as a random factor in all analyses, but site effects are not shown. Columns show results of fixed-effect tests (degrees of freedom, F, P) for population density (“pop. density”) the preceding year, and for rainfall and wind speeds in both summer (October to April) and winter (May to September). Boldface font indicates significant values at P < 0.01.

### Correlates of changes in age structure

Fluctuations in population density were disproportionately due to changes in the abundance (density) of juvenile snakes. After years when population densities increased, a higher proportion of individuals were juveniles; the reverse was true in years when population densities declined (omitting two outliers: F_1,34_ = 10.98, P = 0.002). The proportion of the population comprised of juveniles decreased after years with unusually calm conditions in winter (F_1,31.49_ = 4.38, P < 0.05) but was not significantly linked to other weather measures or to population size in the previous year (all P > 0.17). Reproductive output (proportion of females that were gravid) did not predict age structure in the following year (N = 44, r^2^ = 0.00005, P = 0.89).

### Phenotypic correlates of population size and weather conditions

All of the traits that we measured were affected by wind speeds in winter. After winters with calmer-than-usual conditions, snakes were in better condition (mass relative to length), and females were more likely to be gravid, and to produce larger litters (Table 1). No other factors (including population density in the previous year) were significant, except that snakes tended to be in better body condition after winters with less-than-average rainfall (Table 1).

## Discussion

At each of the three sites that we studied, the population densities of turtle-headed sea snakes varied among years. Much of that variation was due to recruitment; that is, annual differences in the numbers of juvenile snakes. Our analyses identify two correlates (and thus, plausible determinants) of that numerical variation. First, recruitment rates were reduced when population densities were high, consistent with competition for finite food resources (presumably exploitative rather than interference competition, because we have never seen overt behavioural interference among free-ranging snakes). Second, windy conditions in winter enhanced the numbers of juvenile snakes in the population, but reduced body condition as well as the reproductive output of adult snakes.

Our sampling occurred only once per year, precluding us from partitioning changes in abundance into shifts due to reproductive output, survival, and immigration/emigration. Because we sampled only in summer, when females were gravid, we do not know if the juveniles that we captured were the progeny of females from within the population, or had immigrated from elsewhere. Neonates may be more dispersive than older age classes^22,23^, but the high philopatry of individuals (including, young-of-the year snakes), and genetic divergence among sites^24^ suggest that immigration and emigration are unlikely to be important. Hence, the lack of a significant correlation between reproductive output one year and proportion of juvenile snakes the following year suggests that annual changes in abundance of juveniles are not driven by annual changes in fecundity or migration. Instead, the most plausible mechanism involves differential survival: neonate snakes survive at higher rates in some years than others. Such variation has been documented in other tropical snakes (both terrestrial and aquatic) and has been linked to annual variation in prey availability^13,14^.

A combination of density-dependence and stochastic (weather-driven) influences on population density is unsurprising. Finite resources ultimately limit all populations, so that the primary axis of variation in terms of population-regulation may be whether or not a population remains close to that limit versus being kept well below it by stochastic events—so that in the latter case, density-dependence may play little role in driving temporal variation in abundance^1,2^. In turtle-headed sea snakes, the significant statistical signal of density-dependence suggests that populations frequently reach a point at which increased densities reduce average viability of individuals. That process may act by exacerbating competition for a finite food supply (the eggs of demersal-spawning fishes); but could also be mediated by higher rates of pathogen transfer at increased population densities, or if predators are attracted to sites of high prey availability. Our data do not allow us to distinguish among these alternatives; all we can say is that juvenile snakes appear to be disproportionately disadvantaged by increased population density.

Annual variation in snake density and in traits such as body condition and reproductive output was also linked to weather conditions in the year preceding our sampling. Wind speeds are lower during winter than in summer (means of 4.56 vs. 5.58 m/s), creating calm-water conditions that facilitate feeding because snakes can move about more easily, and are not thrown against sharp coral by strong currents. Hence, a calmer winter may allow more time for feeding, resulting in enhanced body condition and reproductive output. In contrast, calm winters appear to decrease survival rates of juveniles. This effect may occur through rendering the snakes more visible to potential predators. Smaller snakes may be more at risk, because they are slower than adults^25^ and are ingestible by a wider range of gape-limited predators^26^. In terrestrial snakes, predators sometimes target smaller individuals^27^. Consistent with the hypothesis that small body size increases the vulnerability of a sea snake, the offspring of most marine snakes are larger than those of terrestrial snakes because (a) offspring size increases with mean adult body size, and adult sea snakes are larger than most terrestrial species^28^; and (b) the offspring of marine snakes are larger relative to adult body size^29^.

Although thermal factors have often been implicated as critical determinants of viability for terrestrial snakes^12,27^, temperatures are buffered by the ocean at our study sites, and vary less from one year to the next than between winter and summer in any given year (annual means 27°C in summer; 23°C in winter; also see^30^). Our preliminary analyses revealed no significant effects of annual thermal variation on snake population densities or other parameters. Although individuals of some species of hydrophiine snakes bask on the surface of the ocean^31^, we have never seen this behaviour in turtle-headed sea snakes; instead, these snakes broach the water surface only briefly when they come up to breathe, swimming down rapidly as soon as they obtain a lungful of air (R. Shine, pers. obs.). That behaviour is consistent with the threat of aerial predation, rather than with behavioural thermoregulation. Snakes tended to be thinner after a winter with unusually heavy rainfall, perhaps reflecting reduced foraging opportunities or reduced breeding by fishes.

The link between weather conditions and reproductive rate accords with the hypothesis that, unlike most snakes, *Emydocephalus* are “income breeders” rather than “capital breeders”^32^. Masunaga et al.^33^ proposed this idea based on dissections of the congeneric species *E. ijimae*, in which most adult females begin vitellogenesis every year, but few eventually ovulate. That is, follicular atresia is common, as expected if females begin with an “optimistic” forecast of the amount of energy they can gather prior to ovulation but abort the development of some of those follicles if food intake falls below the level needed to sustain a large litter^33^. Our data accord well with this scenario, with a female’s “decision” to reproduce, as well as her litter size, influenced by wind strengths over the preceding winter (a primary feeding season for female turtle-headed sea snakes^34^).

The significant density-dependence of population densities in turtle-headed sea snakes predicts that this species should exhibit “K-selected” life-history traits such as delayed maturation, infrequent reproduction and low fecundity^6,35^. That prediction is supported by the biennial reproductive cycle and small litter size of snakes in our study area, but at first sight is inconsistent with relatively early maturation (at two to three years of age^36^). However, the large size at birth for turtle-headed sea snakes (around 300 mm snout-vent length [SVL]) relative to the size at maturation (500 mm SVL^36^) means that ages to sexual maturity cannot be directly compared with those of terrestrial snakes that produce much smaller offspring^29^. That is, sea snakes need to grow less between birth and maturation than do terrestrial snakes; and hence, similar ages at maturation in the two groups^36^ imply a slower growth rate in juvenile sea snakes than in their terrestrial counterparts. Overall, then, our data conform to the scenario of a “K-selected” species whose abundance is regulated at least partly by density-dependence.

Our study provides the first long-term dataset on population fluctuations in hydrophiine snakes, so we cannot compare the dynamics of our turtle-headed sea snake populations with those of other species. Worryingly, however, sea snakes have been reported to exhibit population declines in some areas, even on protected reefs, in some cases leading to local extinctions (with ambiguous causation^37,38^). Given the substantial diversity in life-history traits among hydrophiine species^29,36,39^, population dynamics of sea snakes are likely to be equally diverse, and generalisations therefore elusive. We urgently need research on long-term population dynamics of other species of sea snakes, perhaps using cost-effective methods such as video-camera stations^40^. Until we understand the factors that drive annual variation in the abundance of these marine predators, we will struggle to understand the factors that are driving those widespread declines.

## Methods

### Study site and species

**Figure 3:**
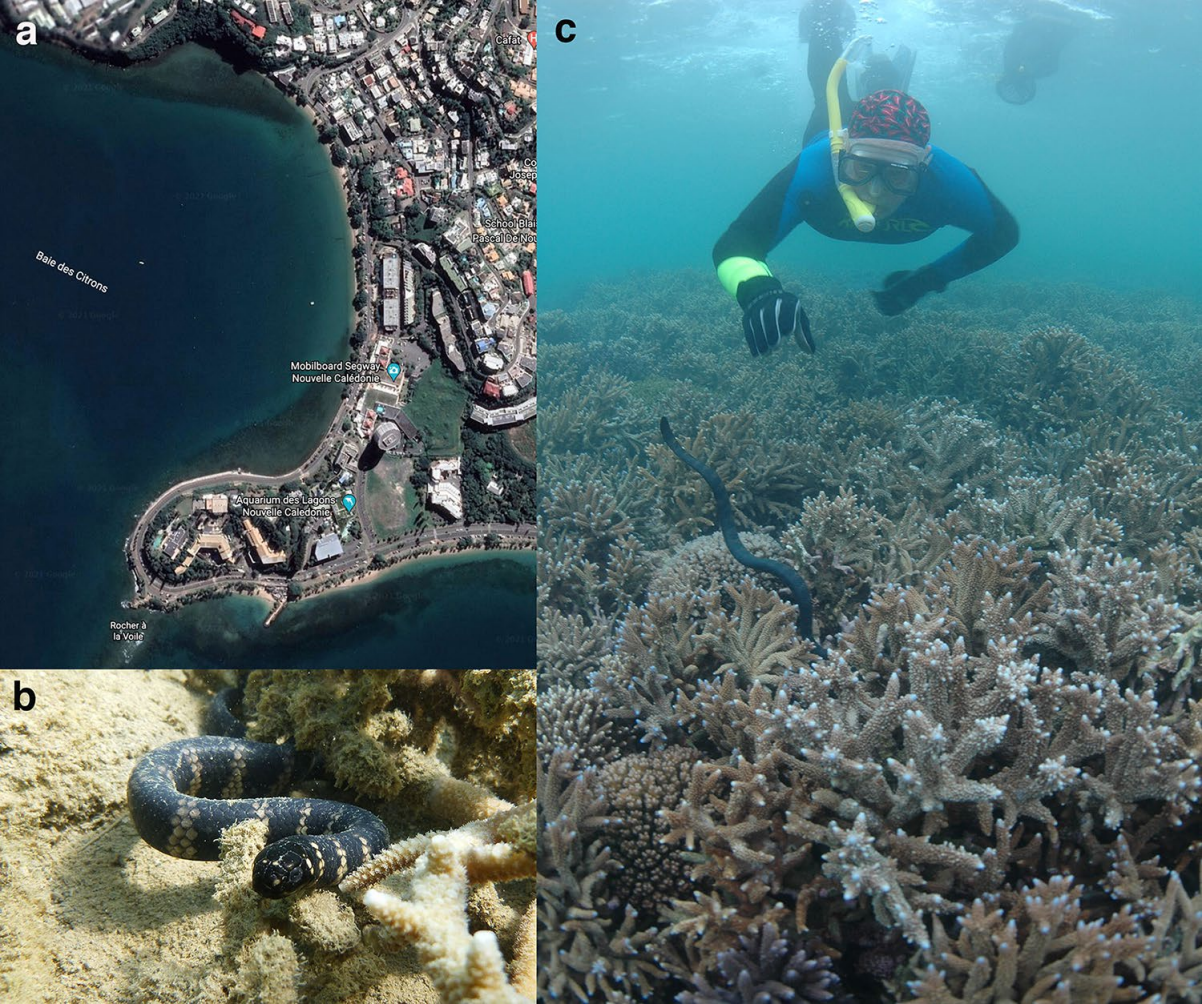
Photographs of (**a**) the study areas in which we conducted mark-recapture studies on sea snakes; (**b**) a turtle-headed sea snake, *Emydocephalus annulatus*; (**c**) capturing a snake during annual surveys. Photographs by Google Earth (**a**), Claire Goiran (**b**), and Pierre Larue (**c**). Map data: Google Earth 2019.

Anse Vata and the Baie des Citrons (22° 16′ S, 166° 26′ E) are adjacent shallow bays beside Noumea, capital city of the Pacific archipelago of New Caledonia (Fig. 3a). Sea snakes are found primarily in coral-reef habitats at either end of each bay, which are separated by sandy substrate^41^. Thus, the turtle-headed sea snakes that we studied in this area form three discrete populations (north end of Anse Vata, north and south ends of Baie des Citrons). These snakes are so highly philopatric that interchange of individuals among the three populations is very rare (despite their proximity: < 1.5 km apart) based on phenotypic traits^42^ and genetic data^24^.

The three study areas that we survey for snakes are each 350 X 20 m in size, extending from the shoreline to about 4 m water depth, over a substrate dominated by live and dead coral, sand, and boulders^43^. Tidal range is 1.5 m^43^. Water temperatures average around 27 °C in midsummer (February) and 23 °C in midwinter (August: https://www.seatemperature.org/australia-pacific/new-caledonia/noumea.htm). Headlands block the often-strong winds, generating relatively calm conditions especially in winter (see below). However, shifts in wind direction and increases in wind speed sporadically produce much rougher conditions; and over the course of our study the sites have intermittently been hit by cyclones and affected by coral bleaching^43^.

Turtle-headed sea snakes (*Emydocephalus annulatus*) belong to the aipysurine lineage of the hydrophiine elapids (“true sea snakes”) and are entirely aquatic. They are relatively short, heavy-bodied snakes (to 800 mm SVL, 480 g; see Fig. 3b) that feed entirely on the demersal eggs of small fishes (blennies, gobies, damselfish); snakes obtain those eggs by scraping against the coralline substrate with their enlarged upper labial scales^34^. Consistent with that diet, the venom glands have atrophied and the venom is weak^44^. Both sexes mature at around 500 mm SVL at two to three years of age, and adult females produce a litter of one to three (usually, two) large (300 mm SVL) offspring on an approximately biennial schedule^36^. Annual survival rates are around 70%, but some individuals live for more than a decade^36^. Mating occurs in winter, and females are gravid from early to late summer, giving birth in May^36^. Males rarely feed during the mating season (in winter) whereas females cease feeding when they are heavily distended by developing embryos (in summer^34^). Known predators of sea snakes include large fishes (especially sharks) and seabirds (especially sea eagles)^45–49^.

### Methods

Every January from 2004 to 2021, we snorkelled through the three study areas to find and capture snakes. Each site was surveyed at least six times per year, over a 2-week period, for 30–60 min each time, by 3–12 divers (Fig. 3c; see^36^ for details). Captured snakes were returned to a nearby laboratory to be measured, weighed and microchipped for individual identification, and then released at the site of capture < 60 min later. Gravid females are easily recognisable by their distended midbodies, and oviductal embryos can be palpated and counted by careful pressure on the snake’s abdomen. Captured snakes were marked by a scale-clip recognisable in the field, allowing us to collect only unmarked snakes on subsequent dives (i.e., each snake was only caught once per year, and our counts do not include multiple records of the same individual within a given year).

The present analysis is based on a total of 2818 capture records over the period 2004–2021 (mean = 156.6 per year, range 101–231). For the first several years we worked only at Anse Vata and the southern end of Baie des Citrons, but we began microchipping snakes at the third site (north end of Baie des Citrons) in 2016. Thus, we have 18 years of data for two sites, but only 6 years of data for the third. All of our statistical analyses (except those for differences among sites) include site as a random factor, to avoid pseudoreplication.

### Independent variables

Jolly-Seber estimates of population sizes for each site in each year were calculated using the POPAN implementation in the MARK software package^50^. Goodness-of-fit tests indicated that the mark-recapture data from each site were appropriate for analysis using Cormack–Jolly–Seber models (all P > 0.06). For each site we constructed a set of candidate models in which parameters for survival (phi), recapture (p) and recruitment (pent) were allowed to vary among years (t) or to remain constant (·). We then selected the best model for each site based on corrected Akaike Information Criteria (AICc) values. At both Anse Vata and Baie des Citrons south the top-ranked model was fully time-dependant (Phi(t) p(t) pent (t); Table 2). At Baie des Citrons north the top model was one in which survival and recapture rates were both constant over time but with time-dependent recruitment (Phi(·) p(·) pent(t); Table 2). Annual estimates of population density for each site were derived from the top-ranked model. Parameter estimates for population sizes, survival, recapture, and recruitment at each site can be found in Supplementary Materials (Appendices 1 and 2).

**Table 2:** Rankings of the top three mark-recapture models for each study site.

Site	Model	AICc	ΔAICc	Parameters	Deviance	− 2 log likelihood
AV	Phi(t) p(t) pent(t)	2593.95	0.00	53	− 2706.37	2482.79
	Phi(t) p(·) pent(t)	2616.82	22.87	36	− 2646.71	2542.45
	Phi(·) p(·) pent(t)	2639.46	45.52	20	− 2590.44	2598.73
BCS	Phi(t) p(t) pent(t)	2175.97	0.00	53	− 1889.63	2063.74
	Phi(t) p(·) pent(t)	2177.92	1.96	36	− 1850.29	2103.08
	Phi(·) p(t) pent(t)	2211.82	35.85	37	− 1818.56	2134.81
BCN	Phi(·) p(·) pent(t)	751.10	0.00	8	− 568.50	734.75
	Phi(t) p(·) pent(t)	758.58	7.48	12	− 569.44	733.80
	Phi(t) p(t) pent(t)	765.37	14.27	17	− 573.42	729.82

For each site a set of models was constructed in which the three parameters (survival (phi), recapture (p) and entry (pent)) were held constant over time (·) or allowed to vary over time (t). Abundance estimates were obtained from the top-ranked model for each site. At both Anse Vata (AV) and the Baie des Citrons south (BCS) the top-ranked model was one in which all three parameters varied over time (Phi(t) p(t) pent(t)). At the Baie des Citrons north (BCN) = the best model was one in which survival and recapture were constant over time but entry varied annually (Phi(·) p(·) pent (t).

To quantify abiotic conditions that might affect snake populations, we first divided the year into summer (October to April, encompassing vitellogenesis and pregnancy) and winter (May to September, the mating season and the first few months of life for neonates^36^). Abiotic factors were calculated separately for each of these seasons, because of the biological differences between them. For example, recruitment might be especially sensitive to weather-driven constraints on feeding or survival during winter (when females feed but males do not; and when neonates are small)^34,36^. A priori, we predicted that the two most important weather variables for snakes would be rainfall (access to freshwater enhances snake body condition^51^) and wind speed (stronger winds create rough sea conditions, constraining shallow-water foraging but hiding snakes from aerial and aquatic predators). We extracted mean monthly values for these variables from the Météo-France climate database for Noumea (http://www.meteo.nc/donnees-publiques/publitheque) and calculated seasonal means from these mean monthly values. With these indices, we can ask whether the direction and degree of change in population density from one year to the next is related to the number of snakes initially present, and/or to weather conditions experienced in the year prior to our January sampling.

### Dependent variables

1. *Annual change in population density.* Quantified by dividing the estimated number in one year by that in the preceding year (Jolly–Seber estimate for Year N divided by that for Year [N − 1]), and calculating the difference between that number and 1.0 (i.e., magnitude of proportional change). To eliminate bias due to proportional data being asymmetric (bounded within the range 0–1 for numbers < 1.0, but able to range from 1 to infinity for numbers above 1), we repeated the analysis with numerator and denominator reversed, to obtain symmetric deviation scores. We then arbitrarily defined all cases of decreases as negative, and all cases of increases as positive^52^. Negative scores reflect decreases in population density, and positive scores show increases; and in both cases, are bounded within the same range (0–1).
2. *Age structure*. Quantified as the proportion of captured snakes that were adults (> 500 mm SVL), and ln-transformed (ln [1 + X]) to improve normality.
3. *Body condition of snakes*. Quantified by taking residual scores from the general linear regression of ln body mass against ln snout-vent length. Positive scores represent snakes that were heavier-than-expected based on their body length; our analyses used means per population per year.
4. *Reproductive frequency*. Quantified as the proportion of adult-sized female snakes (> 500 mm SVL) that were gravid when palpated; our analyses used means per population per year.
5. *Litter size*. Determined by abdominal palpation of females; our analyses used means per population per year.

### Analyses

There has been considerable debate about methods for detecting density-dependence from time-series data on animal abundances^2,4,53^. We used the procedure and code developed by Dennis and Ponciano^21^, to look for density-dependence in the two long-term datasets (i.e., for Anse Vata and for Baie des Citrons south). The procedure compares the goodness of fit of two state-space models fitted to time-series abundance data. The Exponential Growth state-space analysis models time-series as a density-independent process and the Ornstein-Uhlembeck state-space analysis models time-series as density-dependent. A parametric bootstrap likelihood ratio test (based on 1000 bootstrap replicates) is then performed to compare the fits of the density-dependent vs. density-independent models^4^. These analyses were performed in R 4.0.2 using the codes provided as Supplemental Material in Dennis and Ponciano^21^.

We also used a simpler method that allows us to combine data for all three sites, and to compare the effects of density-dependent and stochastic factors within the same analysis. To do so, we conducted linear mixed-model multiple regressions in JMP 15.0. Site (N = 3: Anse Vata, Baie des Citrons north and south) was included as a random factor in all analyses, to account for pseudoreplication (see Table 1). To compare population densities (as estimated by MARK analyses) among sites, we used annual estimates as replicates and conducted an ANOVA with site as a fixed rather than random factor. To look for temporal correlation in the magnitude of annual changes in population densities between the two sites with long-term data, we compared estimated numbers per year for Anse Vata versus Baie des Citrons south. To look for influences on the proportional change in estimated population densities of snakes from one year to the next, we used this parameter as the dependent variable, with the independent variables being population density in the previous year, and mean wind speeds and mean temperatures for summer and winter during the year prior to the survey. To examine the impacts of these same independent variables on traits relevant to recruitment (energy balance and reproductive rate), we repeated the analyses with dependent variables being mean body condition (residual score), proportion of gravid adult females, and mean litter size.

To examine shifts in age structure compared to population change, we used proportion of snakes that were adult in Year [N + 1] as the independent variable, and the proportional change in population density as the dependent variable (i.e., were juvenile snakes under-represented after years in which populations declined?). To clarify effects on age structure, we used proportion of adult snakes as the dependent variable in an ANOVA, with independent variables of population density in the previous year, and wind speeds and rainfall in summer and winter. To evaluate whether reproductive output in one year predicted age structure in the following year, we compared these two variables using correlation analysis.

### Ethical approval

The research was conducted under animal ethics approval 2015/880 (University of Sydney) and permit 3252-17/ARR/DENV (Province Sud, New Caledonia). All procedures involving animals were carried out in accordance with relevant guidelines and regulations (including ARRIVE guidelines).

The person appearing in the photograph in Fig. 3c has given informed consent to publication of their image in this open-access publication.

## Supplementary Information

Supplementary Information.

## Data Availability

Data is available from the Dryad Digital Repository at 10.5061/dryad.qbzkh18hx.
