# Supplementary material for: Population dynamics of the sea snake Emydocephalus annulatus (Elapidae, Hydrophiinae)
Source: Sci Rep. 2021 Oct 19;11:20701. doi: 10.1038/s41598-021-00245-2 (PMC8526600; doi:10.1038/s41598-021-00245-2)
Supplement: Supplementary file 1 — Supplementary Information. [file 41598_2021_245_MOESM1_ESM.pdf]

Supplementary Materials

Population dynamics of the sea snake *Emydocephalus annulatus* (Elapidae, Hydrophiinae)

Richard Shine, Gregory P. Brown & Claire Goiran

Appendix 1. Annual estimates and standard errors of survival (phi), recapture (p) and entry (pent) rates from POPAN analyses at each study site. For sites Anse Vata and Baie des Citrons south all three parameters varied over time (i.e. Phi(t) p(t) pent(t)). At site Baie des Citrons north survival and recapture remained constant over time while entry varied over time (i.e. Phi(.) p(.) pent(t))

Site	year	Phi	p	pent
Anse Vata	2004	0.59 (0.09)	.	0.18 (0.04)
	2005	0.76 (0.08)	0.54 (0.10)	0.06 (0.04)
	2006	0.77 (0.08)	0.39 (0.06)	0.06 (0.03)
	2007	0.63 (0.06)	0.53 (0.06)	0.06 (0.02)
	2008	0.78 (0.07)	0.62 (0.06)	0.06 (0.02)
	2009	0.56 (0.06)	0.68 (0.06)	0.02 (0.01)
	2010	0.43 (0.06)	0.73 (0.07)	0.06 (0.01)
	2011	0.78 (0.09)	0.59 (0.08)	0.02 (0.01)
	2012	0.73 (0.12)	0.62 (0.08)	0.06 (0.02)
	2013	0.66 (0.11)	0.39 (0.08)	0.01 (0.01)
	2014	.	0.48 (0.09)	0.05 (0.02)
	2015	0.54 (0.11)	0.23 (0.05)	0.02 (0.02)
	2016	0.67 (0.16)	0.53 (0.11)	0.06 (0.03)
	2017	.	0.24 (0.08)	0.06 (0.05)
	2018	0.49 (0.12)	0.18 (0.06)	0.07 (0.04)
	2019	0.59 (0.15)	0.39 (0.11)	0.07 (0.03)
	2020	0.20 (0.05)	0.56 (0.14)	0.01 (0.01)
Baie des Citrons south	2004	0.74 (0.09)	.	0.06 (0.02)
	2005	0.87 (0.11)	0.61 (0.10)	0.03 (0.02)
	2006	0.76 (0.10)	0.47 (0.09)	0.04 (0.02)
	2007	0.77 (0.08)	0.52 (0.08)	0.08 (0.02)
	2008	0.93 (0.08)	0.65 (0.08)	0.04 (0.02)
	2009	0.93 (0.16)	0.63 (0.07)	0.23 (0.06)
	2010	0.40 (0.07)	0.27 (0.06)	0.01 (0.03)
	2011	0.78 (0.09)	0.74 (0.07)	0.08 (0.02)
	2012	0.50 (0.07)	0.61 (0.08)	0.06 (0.02)
	2013	0.72 (0.07)	0.49 (0.08)	0.03 (0.02)
	2014	0.88 (0.09)	0.79 (0.06)	0.03 (0.01)
	2015	0.57 (0.09)	0.64 (0.08)	0.02 (0.01)
	2016	0.69 (0.11)	0.52 (0.09)	0.05 (0.02)
	2017	0.86 (0.18)	0.65 (0.10)	0.08 (0.03)

	2018	0.29 (0.07)	0.52 (0.12)	0.02 (0.01)
	2019	0.84 (0.11)	0.66 (0.11)	0.03 (0.01)
	2020	0.60 (0.08)	0.72 (0.10)	0.04 (0.01)
Baie des Citrons north	2016	0.71 (0.03)	0.66 (0.04)	0.15 (0.04)
	2017	0.71 (0.03)	0.66 (0.04)	0.04 (0.03)
	2018	0.71 (0.03)	0.66 (0.04)	0.17 (0.03)
	2019	0.71 (0.03)	0.66 (0.04)	0.13 (0.03)
	2020	0.71 (0.03)	0.66 (0.04)	0.04 (0.02)

Appendix 2. Jolly-Seber abundance estimates, standard errors (SE) and lower and upper 95% confidence limits (CL) for the three study sites. Estimates were obtained using the POPAN implementation in Mark software.

Site	year	Abundance	SE	low 95% CL	high 95%Cl
Anse Vata	2004	57	7.3	44	73
	2005	182	34.7	125	263
	2006	188	27.6	142	251
	2007	198	23.6	157	250
	2008	173	17.6	142	211
	2009	186	18.7	153	226
	2010	123	14.7	97	155
	2011	102	15.3	77	137
	2012	98	14.0	74	130
	2013	119	22.9	82	173
	2014	87	14.8	62	121
	2015	128	24.1	89	184
	2016	88	18.4	59	132
	2017	108	31.6	62	190
	2018	159	42.5	95	266
	2019	137	36.7	81	229
	2020	137	35.6	83	226
	2021	37	5.9	27	51
Baie des Citrons south	2004	44	6.4	33	58
	2005	67	11.5	48	94
	2006	78	13.8	56	110
	2007	80	12.8	58	109
	2008	108	13.7	84	138
	2009	126	15.6	99	161
	2010	250	52.8	166	377
	2011	107	12.7	85	135
	2012	132	18.4	101	174
	2013	100	15.4	74	135
	2014	89	10.2	71	112
	2015	96	13.2	73	125

	2016	69	11.8	50	96
	2017	75	12.2	55	103
	2018	110	25.1	71	172
	2019	41	7.9	28	59
	2020	49	8.8	34	69
	2021	50	6.8	38	65
Baie des Citrons north	2016	117	13.0	94	146
	2017	120	10.2	101	142
	2018	94	8.2	80	112
	2019	107	10.0	90	129
	2020	109	10.3	90	131
	2021	86	9.1	69	105
